# A Dynamic Transcription Factor Signature Along the Colorectal Adenoma-Carcinoma Sequence in Patients With Co-Occurrent Adenoma and Carcinoma

**DOI:** 10.3389/fonc.2021.597447

**Published:** 2021-05-21

**Authors:** Zongfu Pan, Ying He, Wenjuan Zhu, Tong Xu, Xiaoping Hu, Ping Huang

**Affiliations:** ^1^ Department of Pharmacy, Zhejiang Provincial People’s Hospital, People’s Hospital of Hangzhou Medical College, Hangzhou, China; ^2^ Central Laboratory, First Affiliated Hospital, Huzhou University, The First People’s Hospital of Huzhou, Huzhou, China; ^3^ Department of Pathology, First Affiliated Hospital, Huzhou University, The First People’s Hospital of Huzhou, Huzhou, China

**Keywords:** transcription factors, colorectal adenoma-carcinoma sequence, bioinformatics, tissue array, dynamic signature

## Abstract

**Background:**

Colorectal carcinoma (CRC) often arises from benign adenoma after a stepwise accumulation of genetic alterations. Here, we profiled the dynamic landscapes of transcription factors (TFs) in the mucosa-adenoma-carcinoma progression sequence.

**Methods:**

The transcriptome data of co-occurrent adenoma, carcinoma, and normal mucosa samples were obtained from GSE117606. Identification of differentially expressed TFs (DE-TFs) and subsequent function annotation were conducted in R software. Expression patterns of DE-TFs were clustered by Short Time-series Expression Miner software. Thereafter, modular co-expression analysis, Kaplan-Meier survival analysis, mutation profiling, and gene set enrichment analysis were conducted to investigate TF dynamics in colorectal tumorigenesis. Finally, tissue microarrays, including 51 tumors, 32 adenomas, and 53 normal tissues, were employed to examine the expression of significant candidates by immunohistochemistry staining.

**Results:**

Compared to normal tissues, 20 (in adenoma samples) and 29 (in tumor samples) DE-TFs were identified. During the disease course, 28 expression patterns for DE-TFs and four co-expression modules were clustered. Notably, six DE-TFs, DACH1, GTF2IRD1, MEIS2, NR3C2, SOX9, and SPIB, were identified as having a dynamic signature along the colorectal adenoma-carcinoma sequence. The dynamic signature was of significance in GO enrichment, prognosis, and co-expression analysis. Among the 6-TF signature, the roles of GTF2IRD1, SPIB and NR3C2 in CRC progression are unclear. Immunohistochemistry validation showed that GTF2IRD1 enhanced significantly throughout the mucosa-adenoma-carcinoma sequence, while SPIB and NR3C2 kept decreasing in stroma during the disease course.

**Conclusions:**

Our study provided a dynamic 6-TF signature throughout the course of colorectal mucosa-adenoma-carcinoma. These findings deepened the understanding of colorectal cancer pathogenesis.

## Introduction

Colorectal carcinoma (CRC) ranks as the third most frequently diagnosed tumor type and is listed among the top four causes of cancer-related deaths worldwide (1). As has been widely accepted, colorectal carcinoma often arises from benign adenoma, which progresses into malignant cancer as genetic alterations accumulate. This is referred to as the adenoma-carcinoma sequence (2). The average five-year survival rate declines sharply from 90% to 40% once early-stage adenoma develops into malignant forms (3, 4). Early diagnosis and effective management of precursor adenomas are beneficial for improving patient survival. Unfortunately, reliable biomarkers that can predict adenoma progression are still lacking. In order to find novel biomarkers, understanding the molecular changes in the adenoma-carcinoma sequence is needed.

Transcription factors (TFs) act as terminal effectors in carcinogenesis signaling through regulating gene expression, playing an indispensable role in cancer initiation and development (5). Abnormal expression or activity of TFs is associated with colorectal cancer cell growth, invasion, stemness, chemo-resistance, and immune escape (6–9). For instance, STAT3 promoted colorectal cancer cell migration and invasion through activating ZEB1 expression and mediating epithelial-mesenchymal transition (7). Nuclear factor (NF)-κB induced a pro-inflammatory response and contributed to colorectal cancer cell growth (9). Aberrant expression of Wnt transcription factor TCF4 potentiated resistance of colorectal cancer cells to 5-fluororacil-based chemotherapy and X-ray radiotherapy (6). Thus, blocking those TFs are considered as promising therapeutic strategies (5). However, little is known about their dynamic profiling in the mucosa-adenoma-carcinoma progression sequence.

In this study, we analyzed expression data of TFs in patients presenting with concurrent colorectal adenomas and tumors. Those tissues at different evolutionary stages from the same patient could avoid inter-individual variations caused by genetic background, dietary habits, etc. Differentially expressed TFs (DE-TFs) among adjacent mucosae, adenomas, and tumors were identified. Then, their co-expression module networks, relationships with prognosis, mutation landscapes, and related enrichments were comprehensively investigated and visualized.

## Materials and Methods

### Human Specimens

Colorectal adenomas, carcinomas, and adjacent normal paraffin-embedded tissues were retrospectively collected from patients who received surgical resection of colorectal cancer in the First People’s Hospital of Huzhou during 2018, Jan and 2019, Oct. A total of 136 samples were obtained and made into a tissue microarray, including 51 tumors, 32 adenomas, and 53 normal tissues. Informed consent was obtained from all participants.

### Microarray Information

We downloaded RNA expression data of the dataset GSE117606 from Gene Expression Omnibus (GEO, https://www.ncbi.nlm.nih.gov/geo/). This dataset consists of 208 samples from 70 patients, including 69 adenomas, 74 tumors, and 65 mucosa samples. The colorectal tumor samples contain 18 stage I, 24 stage II, 26 stage III, 3 stage IV, and three samples without TNM data. The platform used for these data was Affymetrix HT HG-U133+ PM Array.

### Identification of Differentially Expressed TFs (DE-TFs)

We first screened differentially expressed genes among normal mucosae, adenomas, and tumors in the dataset GSE117606 using the online tool GEO2R (https://www.ncbi.nlm.nih.gov/geo/geo2r/) (10). The False discovery rate (FDR) was automatically calculated by GEO2R and *P* values for multiple testing were adjusted. The thresholds were set as adj.P.Val < 0.05 and |log_2_(Fold change)| ≥ 1. The list of TFs was obtained from the human transcription factor database (http://humantfs.ccbr.utoronto.ca/index.php) and then was intersected by screened differentially expressed genes to get DE-TFs. The Venn diagram was depicted using TBtools (Toolbox for Biologists, Version 0.665) to present the up-regulated and down-regulated DE-TFs. Heatmaps of DE-TFs were drawn by using pheatmap package and RColorBrewer package in the R language software (R3.5.1).

### Expression Manners of DE-TFs During Disease Course

Expression patterns of DE-TFs at different evolutionary stages of CRC (normal mucosa, adenoma, stage I- IV tumor) were analyzed using STEM software, a tool for clustering, comparing, and visualizing short time-series gene expression data (11). FDR was used as the correction method.

### Gene Function and Pathway Enrichment

Gene ontology (GO) and Kyoto Encyclopedia of Genes and Genomes (KEGG) pathway enrichments were conducted for DE-TFs using org.Hs.eg.db package and ClusterProfiler package in R. The FDR < 0.05 was considered statistically significant. The fold changes of DE-TFs enriched in GO and KEGG were visualized using GOplot package and ggplot2 package in R, respectively.

### Determination of Significant DE-TFs in Survival Analysis

The transcriptome sequencing and prognosis data of colon cancers and rectal cancers were obtained from TCGA (The Cancer Genome Atlas, https://tcgadata.nci.nih.gov/tcga/). In R3.5.1, the survival package and survminer package were used to depict the survival curves of each DE-TF in colon cancers and rectal cancers. The best cutoff was calculated and thus the significant DE-TFs (*P* value < 0.05) for survival outcome were determined. Then mutation landscapes of DE-TFs with significant prognostic values and several typical oncogenic genes in CRC were obtained from the cBioPortal database.

### Dynamic Co-Expression Module Analysis and Mutation Landscape of Screened DE-TF Signature

CEMiTool package was used here for dynamic co-expression module analysis in R. This package is based on weighted correlation network analysis (WGCNA) and provides various results including gene modules, co-expression networks, and over representation analysis of biological functions for each module (12). For running CEMiTool, we employed the default gene filter method and parameter settings after importing gene expression matrix, grouping file, c5.bp.v7.0.symbols.gmt enrichment background file, and human protein-protein interaction file. Then, WGCNA package was run to evaluate correlations between obtained modules and DE-TFs. Next, a Venn diagram was drawn to screen DE-TF signatures shared by the abovementioned analysis. Then mutation landscapes of DE-TFs with significant prognostic values and several typical oncogenic genes in CRC were obtained from the cBioPortal database.

### Immunohistochemistry Validation for Significant DE-TFs

The common significant DE-TFs shared by enrichment analysis, survival analysis, and co-expression module analysis in adenomas and tumors were presented by Venn diagram. To validate their expression profiling, we not only obtained immunohistochemistry images from Human Protein Atlas database (https://www.proteinatlas.org) but also performed immunohistochemical staining for our tissue microarray. Briefly, consecutive sections (4 μm) of tissue microarray underwent de-paraffinization and re-hydration, followed by blocking with 5% BSA solution. Thereafter, these sections were incubated with primary antibodies of anti-GTF2IRD1 (Abbkine, 1:100), anti-NR3C2 (Bioss, 1:100), and anti-SPIB (Novus, 1:50) at 4 °C overnight, respectively. After incubation with secondary antibody and treatment with DAB, they were observed under the microscope.

### Gene Set Enrichment Analysis (GSEA)

GSEA is capable of identifying coordinated differential expression of gene sets, providing results of related biological processes and pathways for interested genes (13). In R3.5.1, clusterProfiler package, org.hs.eg.db package, ggplot2 package, and dplyr package were employed to conduct GSEA for SPIB based on its expression. The enrichment background file was c6.all.v7.0.symbols.gmt. A gene set with a FDR < 0.25 was considered significant.

## Results

### DE-TFs in the Colorectal Adenoma-Carcinoma Sequence and Their Expression Patterns

**Figure 1:**
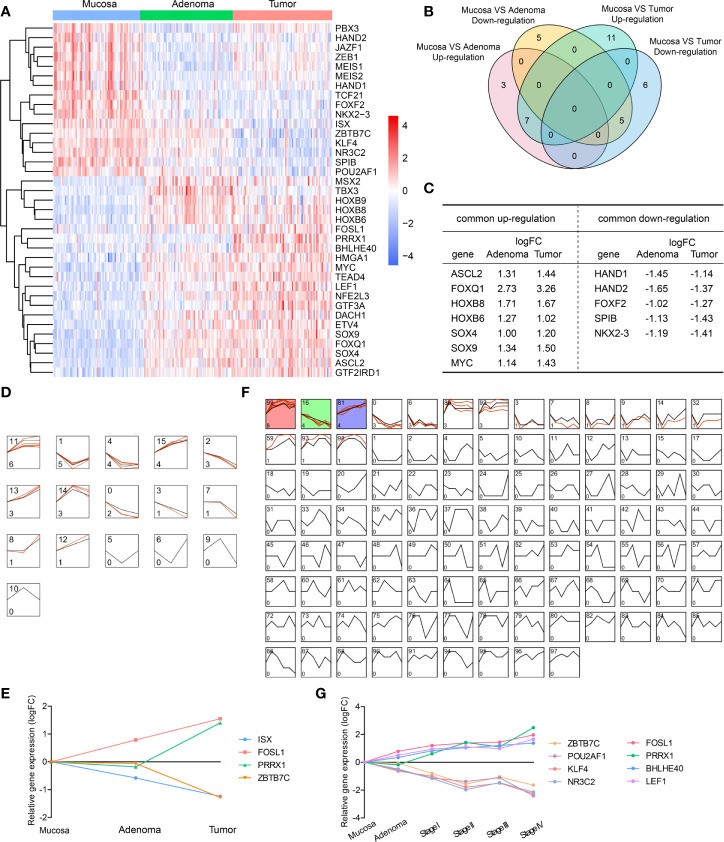
Differentially expressed transcription factors (DE-TFs) at different evolutionary stages of CRC and their expression patterns. **(A)** All up-regulated and down-regulated DE-TFs were hierarchically placed and presented in a heatmap. **(B)** Venn diagram depicted the common DE-TFs shared by adenoma *vs* mucosa, and tumor *vs* mucosa. **(C)** The commonly shared DE-TFs in **(B)** and their fold changes were listed. **(D–G)** Gene expression models in the mucosa-adenoma-tumor sequence **(D)** or mucosa-adenoma-tumor sequence with tumor staging **(F)** were identified by STEM software and representative gene expression patterns was shown in **(E, G)**. Colored models meant a statistically significant number of genes were enriched. The number on the top left corner indicated the serial number of models and the bottom left corner indicated the number of genes enriched.

In patients presenting concurrent colorectal adenoma and carcinoma (data from GEO dataset GSE117606), we analyzed differentially expressed transcription factors (DE-TFs) between adenomas *vs* normal mucosae, and carcinomas *vs* normal mucosae (adj.P.Val < 0.05, |logFC| ≥ 1). The total DE-TFs were presented in a heatmap (**Figure 1A**), consisting of 16 down-regulated DE-TFs and 21 up-regulated DE-TFs. Among them, there were seven commonly up-regulated genes and five commonly down-regulated genes (**Figures 1B, C**).

To investigate the expression profiling of TFs throughout all the evolutionary stages, we used STEM software to depict the dynamic expression patterns in the mucosa-adenoma-tumor sequence. A total of 12 expression models were enriched during the disease progression (**Figure 1D**). For instance, Model 11 had six up-regulated TFs assigned since adenoma stage and five TFs clustered in Model 1 showed specific down-regulation at adenoma stage. Of note, four TFs altered dramatically were presented in **Figure 1E**, namely ISX, FOSL1, PRRX1, and ZBTB7C. In the mucosa-adenoma-tumor (stage I - IV) course, 16 out of 100 presumed Model profiles contained clustered TFs, among which Model 99, 16, and 81 (Models labeled in color) had a statistically significant number of genes enriched (**Figure 1F**). Similar to **Figure 1E**, we obtained another five mostly changed genes (**Figure 1G**), including POU2AF1, NR3C2, and LEF1. These nine genes exhibited stable upward or downward expression patterns during the sequence, suggesting their key roles in potentiating or suppressing CRC initiation and progression.

### Gene Annotation and Pathway Enrichment for DE-TFs

**Figure 2:**
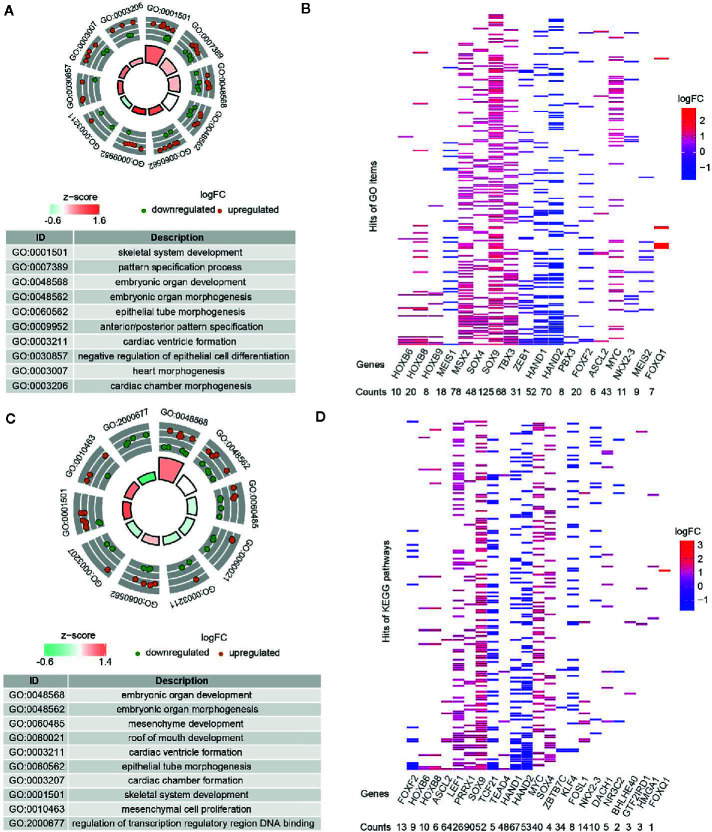
GO-BP (Biological processes) annotation for DE-TFs. **(A)** Top ten enriched GO-BP terms from DE-TFs between adenoma and mucosa were shown in a GOcircle Plot. **(B)** All significantly enriched GO-BP terms from DE-TFs between adenoma and mucosa were displayed in the heatmap. **(C)** Top ten enriched GO-BP terms from DE-TFs between tumor and mucosa were shown in a GOcircle Plot. **(D)** All significantly enriched GO-BP terms from DE-TFs between tumor and mucosa were displayed in the heatmap.

DE-TFs (adenoma *vs* normal and carcinoma *vs* normal) were subjected to gene function annotation and pathway enrichment analysis to uncover their possible biological functions. For DE-TFs between adenoma and normal tissues, the top 10 significantly enriched Gene Ontology Biological Process (GO-BP) terms were displayed in the GO circle plot, including “skeletal system development”, “pattern specification process”, “embryonic organ development”, “embryonic organ morphogenesis”, “epithelial tube morphogenesis”, “anterior/posterior pattern specification”, “cardiac ventricle formation”, “negative regulation of epithelial cell differentiation”, “heart morphogenesis”, and “cardiac chamber morphogenesis” (**Figure 2A**). Similarly, the top 10 significantly enriched GO-BP terms for DE-TFs between carcinoma and normal tissues contained “embryonic organ development”, “embryonic organ morphogenesis”, “mesenchyme development”, “roof of mouth development”, “cardiac ventricle formation”, “epithelial tube morphogenesis”, “cardiac chamber formation”, “skeletal system development”, “mesenchymal cell proliferation”, and “regulation of transcription regulatory region DNA binding” (**Figure 2C**). Notably, quite a lot of DE-TFs were enriched in stemness-related terms, which are well-known to be crucial in potentiating CRC development. **Figures 2B, D** showed the frequency of enriched DE-TFs among the GO-BP terms. Consistently with GO enrichment, the most significantly enriched KEGG pathway was “signaling pathways regulating pluripotency of stem cells” (**Figure S1**), indicating these DE-TFs might be involved in regulatory pathways and processes related to cancer stemness.

### Significant DE-TFs in Survival Analysis

**Figure 3:**
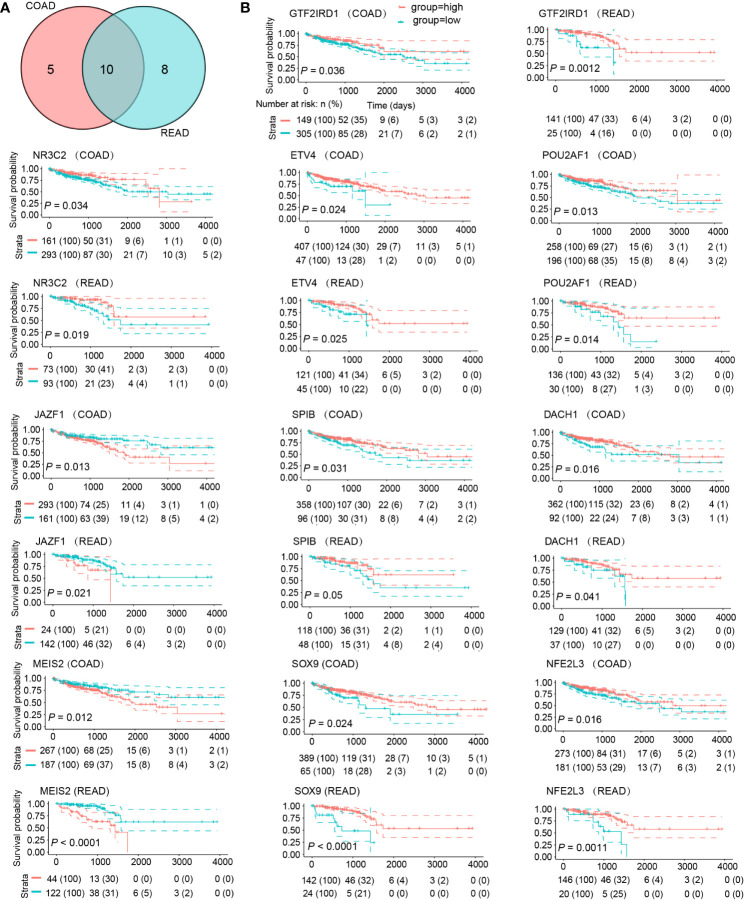
DE-TFs of significance in survival analysis. **(A)** Venn diagram determined ten commonly shared DE-TFs by COAD and READ which possessed significant prognostic value. **(B)** The overall survival curves of these ten DE-TFs in COAD and READ were depicted by Kaplan-Meier method. Patients were grouped into “High” and “Low” depending on the gene expression value with the best cut-off.

To demonstrate the prognostic significance of DE-TFs, we conducted survival analysis using the Kaplan-Meier method in patients with colon adenocarcinoma (COAD) and rectal adenocarcinoma (READ), respectively. We found 23 DE-TFs were statistically significant in prognosis (*P* **<** 0.05), among which 10 DE-TFs were shared by COAD and READ (**Figure 3A**): NR3C2, NFE2L3, JAZF1, ETV4, SPIB, POU2AF1, DACH1, SOX9, MEIS2, and GTF2IRD1. As displayed in **Figure 3B**, high JAZF1 and MEIS2 were associated with poor overall survival, whereas the other eight TFs presented opposite relationships with survival.

### Identification of Dynamic DE-TF Signature Along the Colorectal Adenoma-Carcinoma Sequence

**Figure 4:**
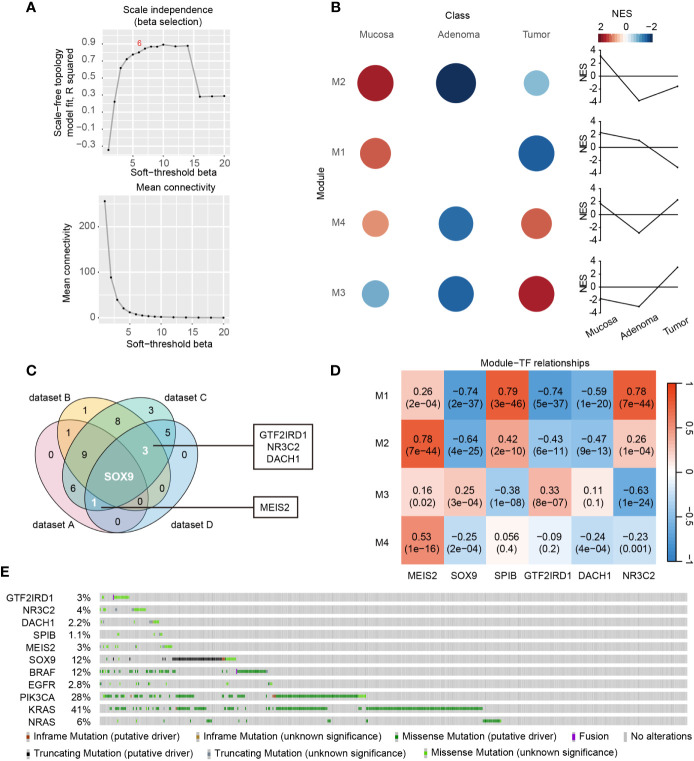
Screening of dynamic DE-TF signature during the disease course. **(A)** Identification of β value based on scale-free topology and mean connectivity in CEMiTool package. **(B)** Significantly enriched modules and the module activity on each class of samples were shown in colored circles. Circle size is in direct proportion to the normalized enrichment score (NES). **(C)** Venn diagram displayed the DE-TFs commonly shared by Dataset A, C, and D, or Dataset B, C, and D. Dataset A, DE-TFs between adenoma and normal mucosa highly enriched in GO-BPs; Dataset B, DE-TFs between carcinoma and normal mucosa highly enriched in GO-BPs; Dataset C, DE-TFs significantly correlated with co-expression Module 1-4; Dataset D, DE-TFs of significance to overall survival; **(D)** The correlations between Module 1 - 4 and six-TF signature genes were determined by WGCNA package. The correlation coefficients and *P* values were displayed in a heatmap. **(E)** Mutation landscapes of six-TF signature genes and several well-known oncogenes, including BRAF, EGFR, PIK3CA, KRAS, and NRAS.

To gain novel insights about DE-TFs-mediated adenoma-tumor evolution, we performed time-course gene co-expression analysis using CEMiTool. The samples clustering showed clear separation among normal mucosa, adenoma, and tumor samples (**Figure S2A**). The β value was automatically chosen as 6 based on an algorithm with a default threshold of R^2^ > 0.8 in CEMiTool (**Figure 4A**), which was assumed to be the best β value to achieve the optimal balance between topology network and connectivity (12). Four significant gene modules were identified by CEMiTool (**Figure S2B**) and their dynamic normalized enrichment score (NES) curves were displayed in **Figure 4B**. Among the three stages, activities of Module 2 and 1 were higher in normal mucosae. Module 3 yielded higher activity in tumors while Module 4 showed the lowest activity in adenomas. Correlations between DE-TFs and each module were analyzed and significant ones (*P* **<** 0.05 and correlation coefficient |r| > 0.5) were shown in **Figure S2C**.

Next, we depicted a Venn diagram to screen dynamic DE-TF signature based on findings in all the abovementioned analyses. Significant DE-TFs shared by GO enrichment, prognosis, and co-expression analysis were identified as meaningful signatures, including GTF2IRD1, NR3C2, DACH1, MEIS2, and SOX9 (**Figure 4C**). Moreover, we found SPIB was significantly down-regulated in both colorectal adenoma and carcinoma, and was a hit of co-expression (dataset C) and prognosis analysis (dataset D) (**Figure S3**). Hence, these six genes were considered as the most meaningful transcriptional signature along the mucosa-adenoma-carcinoma sequence that not only play crucial roles at different stages but also correlate with patient outcome. For example, SPIB and NR3C2 were positively correlated with Module 1 (**Figure 4D**), indicating they are continuously inhibited during the mucosa-adenoma-carcinoma progression. This is in line with their positive role in prognosis and enriched GO terms. Another example is MEIS2 in Module 4. MEIS2 became activated during adenoma-carcinoma transition, which is consistent with its migration promoting role in CRC (14) and its role as a negative outcome predictor. Moreover, the gene set over-representation analysis revealed Module 4 was associated with BPs regarding cell migration, suggesting an oncogenic role of MEIS2 (**Figure S2D**).

Additionally, we obtained the mutation landscape of these six DE-TF signature genes coupled with five frequently mutated genes in CRC, namely BRAF, EGFR, PIK3CA, KRAS, and NRAS (**Figure 4E**). The mutation frequency of six DE-TFs ranged from 1.1% to 12%. Of note, SOX9 possessed the highest mutation rate among the six DE-TFs, and truncating mutation accounted for the majority. For the other five DE-TFs, missense mutation accounted for most mutated cases, indicating their meanings were largely unknown in CRC.

### Immunohistochemistry Validation of Dynamic DE-TF Signature

**Figure 5:**
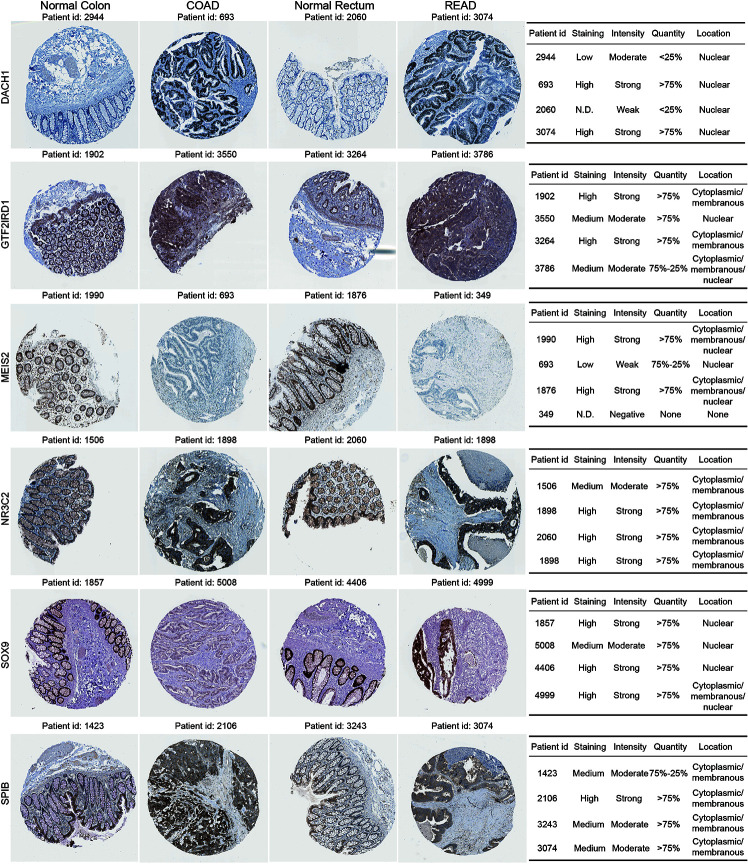
Immunohistochemistry validation for dynamic DE-TF signature in the Human Protein Atlas database. Immunohistochemical images were obtained from the Human Protein Atlas database for six DE-TFs.

We validated protein expression for the dynamic DE-TF signature using immunohistochemistry images from the Human Protein Atlas database (**Figure 5**). Compared to normal tissues, MEIS2 and GTF2IRD1 were down-regulated in both COAD and READ tissues, while DACH1 and SPIB were up-regulated in both. However, enhanced expression of NR3C2 was only observed in COAD and down-regulated SOX9 was only observed in COAD. Additionally, different subcellular locations were noticed. Nuclear localization of SOX9, DACH1, MEIS2, and GTF2IRD1 were observed, which is the usual location for TFs to function. Interestingly, SPIB and NR3C2 were localized outside the nucleus, indicating they might function in distinct ways (**Figure 5**).

**Figure 6:**
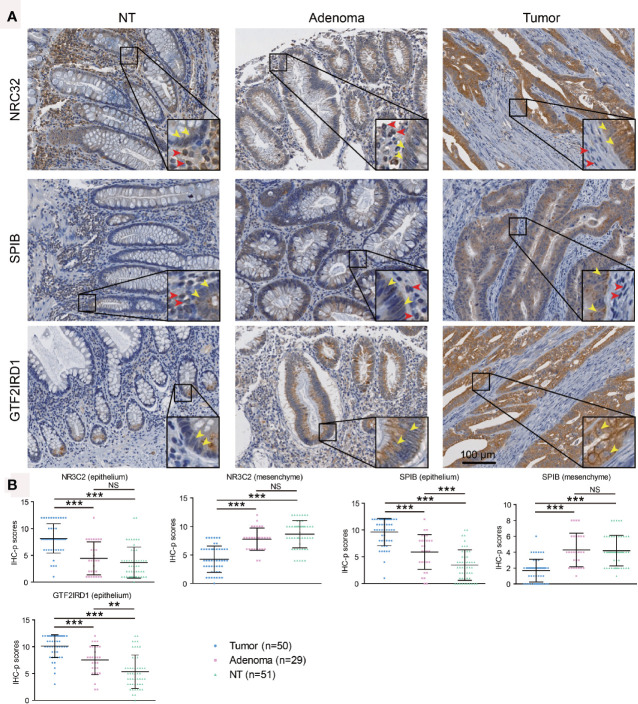
Immunohistochemistry validation for SPIB, NR3C2, and GTF2IRD1 using tissue microarray. **(A)** Representative images showing protein expression in epithelium and stroma. Yellow and red arrows indicated positive staining in epithelium and stroma, respectively. **(B)** Immunohistochemistry scores for expressions of SPIB, NR3C2, and GTF2IRD1. ^**^ indicated *P* < 0.01, ^***^ indicated *P* < 0.001, and NS indicated no significance.

As the roles of SPIB, NR3C2, and GTF2IRD1 are not clear in CRC progression, we further performed immunohistochemistry for them using our tissue microarray. Finally, 50 tumors, 29 adenomas, and 51 normal mucosae were included in our IHC-p score analysis, since tissues on three slides were missing and no adenomatous focus were observed in microscopy on three adenoma slides. Compared with adenoma and normal epithelium, enhanced expression of NR3C2 was observed in epithelial tumor cells (**Figure 6**), which is in line with that in the Atlas database (**Figure 5**). However, NR3C2 expression in stroma showed the opposite tendency to that in epithelium (**Figure 6**), which might explain the decreased NR3C2 gene expression in tumor in **Figure 1G**. In epithelial cells, SPIB expression increased in the sequence of mucosa-adenoma-tumor, which is similar with that in the Atlas database (**Figure 5**) but converse to **Figure 1C**. This inconsistency might be ascribed to the converse expression pattern of SPIB in epithelium and stroma (**Figure 6**). Additionally, GTF2IRD1 expression also increased in the sequence of mucosa-adenoma-tumor.

### SPIB-Based Gene Set Enrichment Analysis (GSEA)

**Figure 7:**
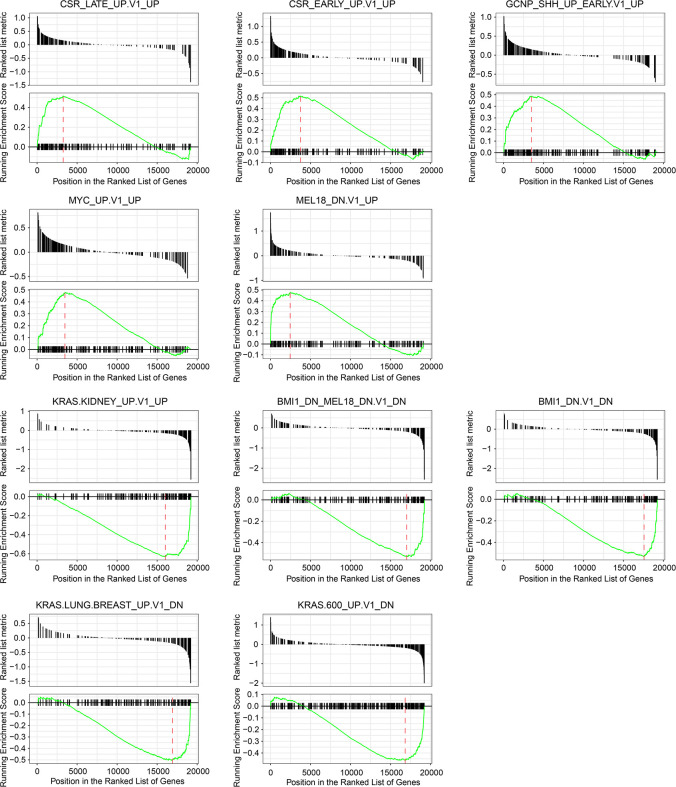
Gene set enrichment analysis based on SPIB expression. Among significantly enriched gene sets, top five up-regulated and down-regulated ones and their corresponding enrichment plots were displayed.

PIB is a transcription factor related to the activity of lymphoid cells and has been reported to be linked with the development of hematopoietic tumors and lung cancer (15). However, its role in colorectal cancer remains unclear. To elucidate its role further, we conducted GSEA using Gene collection C6 (Signatures of oncogenic pathway activation) in the Molecular Signatures Database. Among the markedly enriched gene sets that were associated with SPIB, we listed the top five most up-regulated and down-regulated ones (**Figure 7**, FDR < 0.25). The upper 5 figures showed genes involved in Gene sets “CSR_LATE/EARLY_UP.V1_UP”, “GCNP_SHH_UP_EARLY.V1_UP”, “MYC_UP. V1_UP”, and “MEL18_DN_V1_UP” were markedly up-regulated in populations with high SPIB expression. By contrast, signature genes from Gene set “KRAS_KIDNEY_UP_V1_UP”, “BMI_DN.MEL18_DN.V1_DN”, “BMI1_DN.V1_DN”, “KRAS_LUNG_BREAST_UP.V1_DN”, and “KRAS.600_UP.V1_DN” were significantly repressed in samples with high SPIB expression. Activated “CSR” signatures represented a wound-like phenotype and predicted cancer cell migration and invasion (16). “GCNP” genes were considered as representative hallmarks regarding cancer cell proliferation (17). “MYC”, “BMI1”, “MEL18”, and “KRAS” collections played oncogenic roles in cancer course (18–20). Collectively, SPIB enrichment with these gene sets implied its involvement in various cancer–related biological behaviors.

## Discussion

It is well-known that most, if not all, colorectal cancers originate from adenoma or polyps gradually, which is referred to as the adenoma-carcinoma sequence. Accumulating evidence has been reported to support it, including epidemiological, clinicopathological, and molecular genetic data (21–23). TFs are proven to be pivotal effectors involved in tumor signaling, functioning directly or indirectly in colorectal cancer progression (5). However, the dynamic profiling of TFs in the colorectal adenoma-carcinoma sequence remains obscure. In the present study, we analyzed the TF expression dynamics throughout the mucosa-adenoma-tumor stages using GEO microarray data collected from patients with concurrent colorectal adenomas and tumors, including adjacent mucosae. For the identified DE-TFs, modular co-expression networks, functional enrichment, and prognostic values were assessed to screen key TFs in the adenoma-carcinoma sequence. Finally, we figured out six DE-TFs with good performances in a series of analyses using a Venn diagram: DACH1, GTF2IRD1, NR3C2, MEIS2, SOX9, and SPIB. These six key DE-TFs were considered as dynamic TF signature throughout the adenoma-carcinoma sequence.

The transition from adenoma to carcinoma is based on dynamic changes in molecular profiles, including the alteration of TFs. The close relationships between these six DE-TFs with Module 1-4 further validated their dynamic changes in the mucosa-adenoma-carcinoma sequence. For example, dynamic down-regulation of NR3C2 in the mesenchyme was consistent with activity inhibition in Module 1 during disease course. In consideration of the significant enrichment Module1 with immune responses, NR3C2 was suggested to play a role in the tumor immune microenvironment. Although down-regulation of NR3C2 was reported in COAD and its high expression predicted good prognosis (24), we found the distinct expression trend of NR3C2 in the epithelium and mesenchyme, indicating further experiments are needed to explore their exact function. By contrast, SOX9 was elevated along the sequence and negatively correlated with Module 1. SOX9 has been demonstrated to regulate resistance to T-cell-mediated killing in melanoma cells through CEACAM1, a pivotal protein in crosstalk between cancer cells and immune environment (25). Hence, the role of SOX9 in CRC immunity is also worthy of attention.

Further GO-BP annotation revealed these six DE-TFs enriched with not only embryonic stem cells but also epithelial differentiation. Similarly, they significantly enriched with pathways regarding stem cell pluripotency and cancer. As is known, DACH1 expression was aberrantly augmented in LGR5^+^ intestinal stem cells. It was revealed to predetermine the stem cell proliferation and maintenance in CRC through suppressing BMP signaling (26). Consistently, in our case, DACH1 protein expression was upregulated in the COAD and READ and was highly enriched in stem cell-related GO terms, indicating DACH1 might maintain the stemness of crypt base cells and promote normal epithelium-adenoma-carcinoma progression. In addition, MEIS2 was demonstrated to be hypermethylated and down-regulated in CRC stem cells, playing a prominent role in stemness maintenance and chemotherapy resistance. As for SOX9, it is widely known as a master regulator of cell fate through affecting multiple developmental pathways including differentiation and stemness. For example, it induced and maintained stem-like phenotypes *via* SOX9/LncRNA FARSA-AS1/SOX9 loop activation (27). These reports support the feasibility of our screening strategy.

In survival analysis, MEIS2 acted as a negative prognostic factor while the other five TFs performed as positive prognostic factors both in COAD and READ. For instance, NR3C2 presented as a favorable CRC prognosis factor in literature (24), which is in line with our analysis, though its function in colorectal cancer is still unclear. However, controversial biological functions were also observed for some of them. GTF2IRD1 is an example. Evidence unveiled its oncogenic role and high GTF2IRD1 expression predicted poor outcome in CRC (28), while its high expression was correlated with long survival time in our study, indicating further validation studies are needed. In addition, we identified two new potential prognostic factors, POU2AF1 and JAZF1, whose roles in CRC prognosis are largely unknown.

Notably, SPIB, though few experimental studies described its detailed function and mechanism in colorectal cancer evolution, was identified as a common transcriptional hallmark shared by precancerous adenoma and tumor stages in our study. SPIB is a member of the ETS family which is associated with malignant transformation. SPIB was previously reported to potentiate early mesenchymal invasion and epithelial cell metastasis by repressing CLDN2 nuclear transcription in lung cancer (15). In CRC, SPIB was demonstrated to correlate with CRC incidence risk and could act as a prognostic factor elsewhere (29, 30) In our case, SPIB expression increased in epithelium but decreased in mesenchyme in the sequence of normal mucosa-adenoma-tumor. This expression pattern indicated distinct roles of SPIB in CRC epithelial cells and tumor microenvironment. Furthermore, GSEA analysis indicated SPIB could be involved in CRC cell proliferation, migration, and invasion, which needs further experimental validation.

## Conclusions

In conclusion, we provided the first dynamic landscape of TFs throughout the mucosa-adenoma-carcinoma progression course for colorectal cancer. Six DE-TFs with prognostic value were identified as the most significant transcriptional signature, among which SPIB and NR3C2 are two novel ones. In addition, these DE-TFs were tightly correlated with biological processes and pathways related to immune response, cancer cell stemness, and genetic mutations. Further studies including foundational and multi-omics investigations are needed to comprehensively understand their roles in the pathogenesis of colorectal cancer.

## Data Availability Statement

The original contributions presented in the study are included in the article/**Supplementary Material**. Further inquiries can be directed to the corresponding authors.

## Ethics Statement

The studies involving human participants were reviewed and approved by Ethics committee of First People’s Hospital of Huzhou. The patients/participants provided their written informed consent to participate in this study.

## Author Contributions

PH, ZP, and YH designed the study. ZP, TX, and XH performed data analysis and prepared figures. YH wrote the manuscript with help from all authors. YH and WZ collected the clinical samples and conducted immunohistochemistry experiments. PH, ZP, and YH revised the manuscript. All authors contributed to the article and approved the submitted version.

## Funding

This work was funded by the National Natural Science Foundation, People’s Republic of China (Nos. 81802673, 81970570), Natural Science Foundation of Zhejiang Province (No. LQ20H160011), Chinese Medicine Research Program of Zhejiang Province (No. 2021ZA006), Zhejiang Medical and Health Science and Technology Project (Nos. 2019KY208, 2020KY940, 2021KY056), and the Huzhou Science and Technology Foundation Project (No.2019GZ37).

## Conflict of Interest

The authors declare that the research was conducted in the absence of any commercial or financial relationships that could be construed as a potential conflict of interest.

## Abbreviations

COAD, colon adenocarcinoma; CRC, colorectal carcinoma; DE-TFs, differentially expressed TFs; FDR, false discovery rate; GSEA, gene set enrichment analysis; GO, gene ontology; KEGG, Kyoto encyclopedia of genes and genomes; NES, normalized enrichment score; READ, rectal adenocarcinoma; STEM, short time-series expression miner; TFs, transcription factors; WGCNA, weighted gene co-expression network analysis.
