# Supplementary material for: A Dynamic Transcription Factor Signature Along the Colorectal Adenoma-Carcinoma Sequence in Patients With Co-Occurrent Adenoma and Carcinoma
Source: Front Oncol. 2021 May 21;11:597447. doi: 10.3389/fonc.2021.597447 (PMC8176860; doi:10.3389/fonc.2021.597447)
Supplement: Supplementary file 1 [file DataSheet_1.docx]

**Supplemental figures**


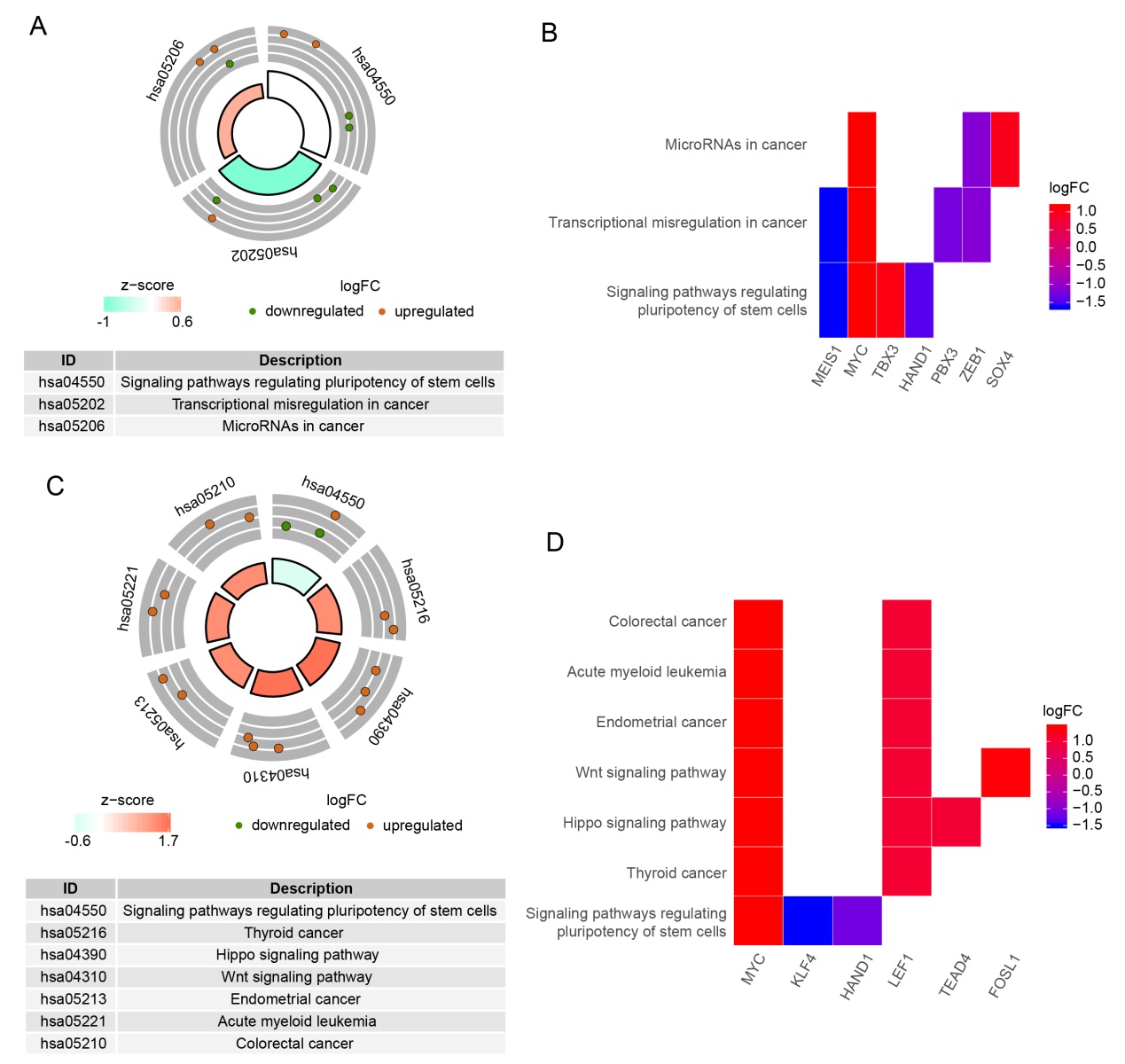


Fig. S1 Kyoto Encyclopedia of Genes and Genomes (KEGG) pathway enrichment for DE-TFs. (A) Significantly enriched KEGG pathways from DE-TFs between adenoma and mucosa were showed in a GOcircle plot. (B) Heatmap for significantly enriched pathways from DE-TFs between adenoma and mucosa. (C) Significantly enriched KEGG pathways from DE-TFs between tumor and mucosa were showed in a GOcircle plot. (D) Heatmap for significantly enriched pathways from DE-TFs between tumor and mucosa.


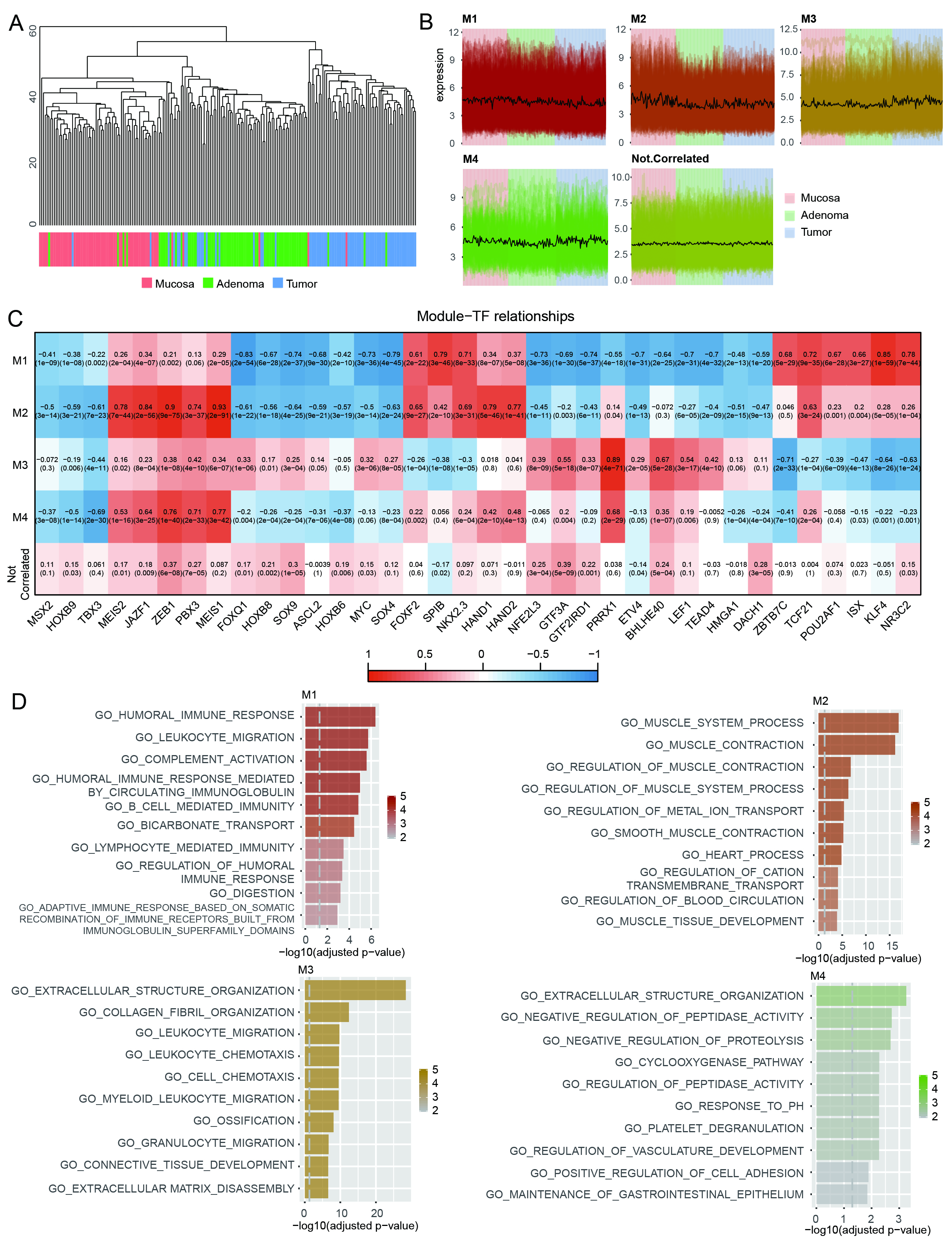


Fig. S2 Gene co-expression modules and their correlations with DE-TFs. (A) Clustering analysis revealed good separation among mucosa, adenoma and tumor samples. (B) Gene expression profile plots of Module 1 - 4 and the uncorrelated module. Black lines indicated the mean expression of all genes inside this module. (C) The correlations between Module 1 - 4 and DE-TFs were determined by WGCNA package. The correlation coefficients and *P* values were displayed in a heatmap. (D) Over representation analysis of Module 1 - 4. Significantly enriched GO-BP terms were listed.


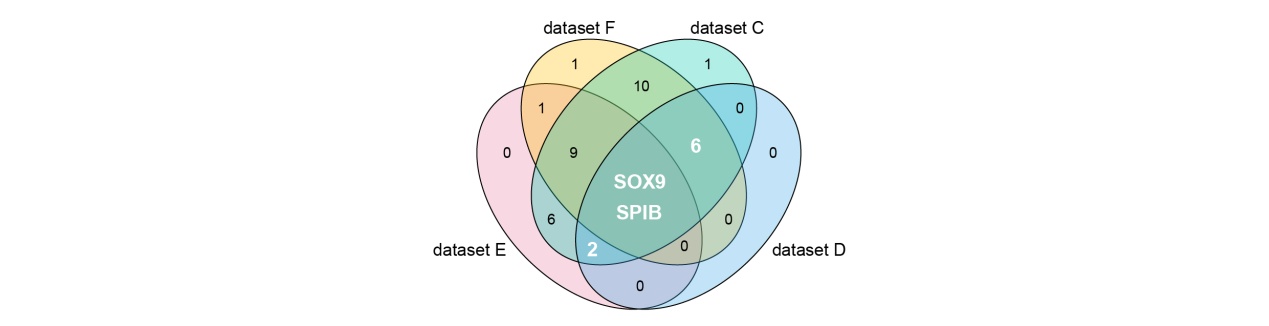
Fig. S3 Screening of significant DE-TF signature. Venn diagram displayed the DE-TFs commonly shared by Dataset E, C, and D, or Dataset F, C, and D. Dataset E, DE-TFs between adenoma and normal mucosa; Dataset F, DE-TFs between carcinoma and normal mucosa; Dataset C, DE-TFs significantly correlated with co-expression Module 1-4; Dataset D, DE-TFs of significance to overall survival.
